# Supplementary material for: The Early Oxidative Stress Induced by Mercury and Cadmium Is Modulated by Ethylene in Medicago sativa Seedlings
Source: Antioxidants (Basel). 2023 Feb 22;12(3):551. doi: 10.3390/antiox12030551 (PMC10045221; doi:10.3390/antiox12030551)
Supplement: Supplementary file 1 [file antioxidants-12-00551-s001.zip › antioxidants-2201463-supplementary.pdf]

Supplementary information to the article:

Methods S1:

Additional information of growing conditions and enzymatic activities.

Supplementary Tables:

Table S1. Root biothiols concentration (nmol g^{-1} FW) of alfalfa seedlings treated with Cd or Hg (0, 3, 10 y 30 μM) for 3, 6 y 24 h.

Table S2. Biothiols concentration (nmol g^{-1} FW) in cotyledons of alfalfa seedlings treated with Cd or Hg (0, 3, 10 y 30 μM) for 3, 6 y 24 h.

Table S3. Influence of the ethylene signalling inhibitor methylene cyclopropene (1-MCP) on biothiols concentration (nmol g^{-1} FW) in roots of alfalfa seedlings treated with 30 μM Cd or 3 μM Hg for 6 y 24 h.

Supplementary Figures:

Figure S1. Growth of alfalfa seedlings grown in the *microscale* system with Cd or Hg (0, 3, 10, 30 μM) for 3, 6 and 24 h.

Figure S2. Cotyledons redox enzymatic activities determined *in gel* after ND-PAGE, of alfalfa seedlings treated with 0, 3, 10 and 30 μM Hg or Cd for 3, 6 and 24 h.

Figure S3. Biothiols' chromatograms of extracts obtained from roots of alfalfa seedlings treated with Hg and Cd (0, 3, 10 and 30 μM) for 24 h.

Methods S1

Plant material and hydroponic system

Seeds of *Medicago sativa* var. Aragon were sterilized by immersion in 5% sodium hypochlorite for 5 min. After excess bleach washing with sterile deionised H_2O, seeds were maintained at 4°C for 24 h, and then germinated in complete darkness on 1.5% (w/v) agar in sterile Petri plates at 28°C for 24 h. Homogeneous seedlings were transferred to a home-made *microscale* hydroponic system [1], containing 150 mL of Murashige-Skoog (MS) nutrient solution. After 24 h acclimation to the growth chamber environmental conditions (16 h light at 25°C, 8 h darkness at 18ºC), seedlings were grown in MS supplemented with $CdCl_2$ and $HgCl_2$ (3, 10 and 30 μM) for 3, 6 and 24 h. In the ethylene signalling inhibition experiments with 1-methylecyclopropene (1-MCP), alfalfa seedlings were grown in identical conditions, but were pre-incubated with 10 μM 1-MCP 24 h prior to metal exposure. 1-MCP was also added to the corresponding growing media during the treatments with Hg (3 μM) and Cd (30 μM). Seedling's length was measured, cotyledons and roots were separated, and snap frozen in liquid nitrogen and stored at -80 °C until analysis.

Redox enzymatic activities *in gel*

Enzymatic extracts were prepared with 0.5 g of frozen material by homogenisation in an ice-cooled mortar and pestle with 1 mL of extracting mixture (10 mL 30 mM MOPS at pH 7.5, 5 mM $EDTA-Na_2$, 10 mM DTT, 10 mM ascorbic acid, 0.6% PVP, 10 μM PMSF and protease inhibitor cocktail). Cell debris were separated by centrifugation (14000 x g) at 4 °C for 15 min, and the supernatant was stored at -80 °C in single-use aliquots of 100 μL. Bio-Rad Protein Assay was used to determine protein concentration in extracts. Protein loading was re-adjusted using denaturing polyacrylamide gel electrophoresis (SDS-PAGE) and Coomassie blue staining, prior

to *in gel* enzymatic activity staining after non-denaturing polyacrylamide gel electrophoresis (ND-PAGE), using conventional settings [2].

GR (EC 1.6.4.2) activity was detected in ND-PAGE (10% polyacrylamide) loaded respectively with 15 μg and 5 μg protein of shoot and root samples, incubated in 250 mM Tris-HCl (pH 7.5), supplemented with trizolyl blue tetrazolium bromide 0.2 mg/mL, 2.6-dichlorophenol indophenol 0.2 mg/mL, 0.5 mM NADPH and 3.5 mM oxidized glutathione (GSSG) [2]. APX (EC 1.11.1.11) activity was determined after ND-PAGE (10% polyacrylamide) loading 5 μg protein. Gels were incubated with 4 mM Na-ascorbate and 2 mM H_2O_2 in 50 mM Na-phosphate buffer at pH 7.0 for 20 min. The presence of APX was detected with a solution of 0.5 mM nitroblue tetrazolium (NBT) and 10 mM TEMED in 50 mM Na-phosphate buffer at pH 7.8 [1]. NADPH oxidase (EC 1.6.3.1) was detected after ND-PAGE (10% polyacrylamide) loaded with 10 μg protein, incubated in 250 mM Tris-HCl buffer (pH 7.5) supplemented with 0.5 mg/mL NBT, 0.2 mM NADPH, 4 mM $CaCl_2$ and 0.2 mM $MgCl_2$ [3].

The pattern of POX isoforms was obtained after isoelectric focusing (IF) ND-PAGE (6.5% acrylamide), using ampholytes that covered the 3.0-10.0 pH range, with buffers composition and electrophoresis conditions described by Ros-Barceló et al. [4]. Protein loading was 15 μg and 5 μg of shoot and root, respectively. POX activity was revealed by incubating the gels in a solution of 100 mM 4-methoxy-naphtol and 10 mM H_2O_2 in 50 mM Na-acetate buffer at pH 5.0.

References

1. Ortega-Villasante, C., Hernández, L.E., Rellán-Álvarez, R., Del Campo, F.F., Carpena-Ruiz, R.O. Rapid alteration of cellular redox homeostasis upon exposure to cadmium and mercury in alfalfa seedlings. *New Phytol*. **2007**, *176*, 96-107. https://doi.org/10.1111/j.1469-8137.2007.02162.x.
2. Sobrino-Plata, J.; Ortega-Villasante, C.; Flores-Cáceres, M.L.; Escobar, C.; Del Campo, F.F.; Hernández, L.E. Differential alterations of antioxidant defenses as bioindicators of mercury and cadmium toxicity in alfalfa. *Chemosphere* **2009**, *77*, 946–954. https://doi.org/10.1016/j.chemosphere.2009.08.007
3. Sobrino-Plata, J.; Meyssen, D.; Cuypers, A.; Escobar, C.; Hernández, L.E. Glutathione is a key antioxidant metabolite to cope with mercury and cadmium stress. *Plant Soil* **2014**, *377*, 369–381. https://doi.org/10.1007/s11104-013-2006-4.
4. Ros-Barceló, A., Pomar, F., Ferrer, M.A.., Martínez, P., Ballesta, M.C., Pedreño, M.A. In situ characterization of a NO-sensitive peroxidase in the lignifying xylem of *Zinnia elegans*. *Physiol. Plant*. **2002,** *114***,** 33-40. https://doi.org/10.1034/j.1399-3054.2002.1140106.x

Supplementary Table S1. Root biothiols concentration (nmol g^{-1} FW) of alfalfa seedlings treated with Cd or Hg (0, 3, 10 y 30 μM) for 3, 6 y 24 h. Different upper script letters denote significant differences between treatments with $p < 0.05$. In parenthesis, it is shown the abundance (%) of each biothiol type (Cys, cysteine; GSH, glutathione; hGSH, homoglutathione; PCs, phytochelatins) relative to the total amount of biothiols per treatment.

		**Treatment**	**BIOTHIOLS**			
			**Cys**	**GSH**	**hGSH**	**PCs**
**Cadmium**	**3 h**	Control	70.4^{a} ± 8.3 (39)	48.0^{a} ± 5.4 (27)	60.1^{a} ± 7.1 (34)	n.d.
		3 μM	40.6^{c} ± 4.9 (36)	35.2^{b} ± 2.5 (31)	36.9^{b} ± 6.9 (33)	n.d.
		10 μM	50.8^{b} ± 4.6 (39)	35.5^{b} ± 1.6 (27)	42.8^{b} ± 3.2 (33)	n.d.
		30 μM	56.8^{b} ± 7.0 (43)	32.5^{b} ± 1.8 (25)	41.5^{b} ± 5.8 (32)	n.d.
	**6 h**	Control	76.2^{a} ± 3.4 (40)	43.2^{a} ± 3.2 (23)	70.4^{a} ± 4.6 (37)	n.d.
		3 μM	65.9^{b} ± 2.1 (43)	33.7^{b} ± 2.3 (22)	52.3^{b} ± 2.3 (34)	n.d.
		10 μM	70.7^{ab} ± 4.8 (47)	32.5^{b} ± 3.3 (21)	48.1^{b} ± 2.6 (32)	n.d.
		30 μM	77.8^{a} ± 1.7 (50)	35.0^{b} ± 2.9 (22)	44.2^{b} ± 4.9 (28)	n.d.
	**24 h**	Control	46.4^{a} ± 3.5 (25)	49.2^{a} ± 1.6 (27)	88.1^{a} ± 1.1 (48)	n.d.
		3 μM	33.2^{b} ± 2.8 (30)	33.0^{b} ± 2.8 (29)	46.1^{b} ± 1.3 (41)	< 10* (< 5)
		10 μM	42.8^{ac} ± 1.8 (23)	35.6^{b} ± 2.9 (19)	59.5^{c} ± 1.4 (33)	44.9^{d} ± 4.6 (25)
		30 μM	41.7^{c} ± 2.4 (19)	48.0^{a} ± 2.6 (22)	58.0^{c} ± 3.2 (26)	74.7^{d} ± 6.5 (34)
**Mercury**	**3 h**	Control	71.1^{a} ± 14.2 (43)	32.0^{a} ± 6.4 (19)	62.6^{a} ± 2.3 (38)	n.d.
		3 μM	62.4^{a} ± 13.7 (47)	28.3^{a} ± 7.7 (21)	41.1^{b} ± 10.9 (31)	n.d.
		10 μM	74.2^{a} ± 7.4 (56)	20.6^{b} ± 2.7 (16)	38.0^{b} ± 4.4 (29)	n.d.
		30 μM	70.1^{a} ± 2.5 (47)	34.5^{a} ± 2.1 (23)	44.2^{b} ± 8.3 (30)	n.d.
	**6 h**	Control	76.2^{a} ± 3.4 (40)	43.2^{a} ± 4.2 (23)	70.4^{a} ± 4.4 (37)	n.d.
		3 μM	74.5^{a} ± 4.6 (50)	25.0^{b} ± 4.6 (17)	48.7^{b} ± 3.2 (33)	n.d.
		10 μM	59.9^{b} ± 13.8 (50)	24.0^{b} ± 1.1 (20)	36.1^{c} ± 2.1 (30)	n.d.
		30 μM	54.3^{b} ± 10.8 (75)	18.1^{b} ± 2.4 (25)	< 10* (< 5)	n.d.
	**24 h**	Control	50.2^{a} ± 7.3 (29)	41.3^{a} ± 5.4 (24)	80.5^{a} ± 6.8 (47)	n.d.
		3 μM	43.9^{a} ± 5.6 (21)	26.6^{ab} ± 2.9 (13)	54.4^{b} ± 6.6 (26)	84.4d ± 8.2 (40)
		10 μM	27.9^{b} ± 4.2 (23)	23.5^{b} ± 3.9 (19)	33.0^{c} ± 3.6 (27)	37.1d ± 1.0 (31)
		30 μM	< 10*	< 10*	< 10*	< 10*

*A peak was detected, but concentration was below 10 nmol g^{-1} FW.

n.d.: not detected.

Supplementary Table S2. Biothiols concentration (nmol g^{-1} FW) in cotyledons of alfalfa seedlings treated with Cd or Hg (0, 3, 10 y 30 µM) for 3, 6 y 24 h. Different upper script letters denote significant differences between treatments with $p < 0.05$. In parenthesis, it is shown the abundance (%) of each biothiol type (Cys, cysteine; GSH, glutathione; hGSH, homoglutathione; PCs, phytochelatins) relative to the total amount of biothiols per treatment.

		Treatment	BIOTHIOLS			
			Cys	GSH	hGSH	PCs
Cadmium	3 h	Control	85.8^a ± 10.1 (38)	46.5^a ± 2.7 (20)	95.0^a ± 6.4 (42)	n.d.
		3 µM	96.4^a ± 18.1 (40)	40.3^a ± 15.1 (17)	105.8^a ± 20.9 (44)	n.d.
		10 µM	117.3^a ± 23.3 (40)	51.5^a ± 18.6 (18)	122.9^a ± 15.8 (42)	n.d.
		30 µM	103.4^a ± 15.8 (38)	56.9^a ± 14.0 (21)	110.1^a ± 21.5 (41)	n.d.
	6 h	Control	108.9^a ± 9.4 (33)	48.2^a ± 8.2 (15)	171.9^a ± 8.6 (52)	n.d.
		3 µM	105.7^a ± 8.7 (34)	38.1^a ± 4.8 (12)	166.3^a ± 15.3 (54)	n.d.
		10 µM	105.6^a ± 14.8 (33)	42.4^a ± 6.7 (13)	174.9^a ± 18.9 (54)	n.d.
		30 µM	118.6^a ± 15.9 (38)	38.7^a ± 2.1 (13)	151.4^a ± 12.5 (49)	n.d.
	24 h	Control	85.7^a ± 8.0 (28)	46.2^a ± 5.2 (15)	171.9^a ± 14.6 (57)	n.d.
		3 µM	86.2^a ± 5.1 (30)	37.1^a ± 2.8 (13)	166.3^a ± 10.3 (57)	n.d.
		10 µM	97.7^a ± 5.9 (31)	41.4^a ± 3.7 (13)	174.9^a ± 10.9 (57)	n.d.
		30 µM	100.8^a ± 11.2 (35)	38.7^a ± 2.1 (13)	151.4^a ± 11.5 (52)	n.d.
Mercury	3 h	Control	122.7^a ± 14.0 (37)	60.7^a ± 2.5 (18)	150.6^a ± 5.5 (45)	n.d.
		3 µM	109.8^a ± 17.2 (34)	54.9^a ± 4.6 (17)	159.6^a ± 7.5 (49)	n.d.
		10 µM	111.9^a ± 18.4 (36)	58.4^a ± 5.5 (19)	141.5^a ± 6.4 (45)	n.d.
		30 µM	101.7^a ± 13.9 (34)	52.7^a ± 5.1 (17)	148.1^a ± 11.3 (49)	n.d.
	6 h	Control	109.0^a ± 9.4 (33)	48.2^a ± 8.2 (15)	171.9^a ± 7.9 (52)	n.d.
		3 µM	114.1^a ± 8.0 (34)	52.6^a ± 10.3 (16)	168.1^a ± 9.9 (50)	n.d.
		10 µM	117.7^a ± 11.5 (33)	61.8^a ± 7.0 (17)	175.2^a ± 19.5 (49)	n.d.
		30 µM	110.8^a ± 14.8 (33)	59.7^a ± 8.1 (18)	165.9^a ± 7.8 (49)	n.d.
	24 h	Control	130.2^a ± 34.1 (46)	37.0^a ± 18.3 (13)	116.2^a ± 28.5 (41)	n.d.
		3 µM	120.4^a ± 8.5 (44)	32.1^a ± 3.3 (12)	121.9^a ± 17.2 (44)	n.d.
		10 µM	115.0^a ± 20.9 (41)	34.5^a ± 5.7 (12)	131.1^a ± 21.8 (47)	n.d.
		30 µM	126.2^a ± 11.6 (45)	34.1^a ± 5.1 (12)	122.5^a ± 18.6 (43)	n.d.

*A peak was detected, but concentration was below 10 nmol g^{-1} FW.

n.d.: not detected.

Supplementary Table S3. Influence of the ethylene signalling inhibitor methylene cyclopropene (1-MCP) on biothiols concentration (nmol g^{-1} FW) in roots of alfalfa seedlings treated with 30 µM Cd or 3 µM Hg for 6 y 24 h. A set of seedlings were pre-incubated with 10 µM 1-MCP for 24 h prior to the exposure to Cd or Hg. Different upper script letters denote significant differences between treatments with $p < 0.05$. In parenthesis, it is shown the abundance (%) of each biothiol type (Cys, cysteine; GSH, glutathione; hGSH, homoglutathione; PCs, phytochelatins) relative to the total amount of biothiols per treatment.

	Treatment	BIOTHIOLS			
		**Cys**	**GSH**	**hGSH**	**PCs**
**6 h**	Control	$36.9^{a} \pm 1.6$ (21)	$35.7^{a} \pm 2.3$ (21)	$100.3^{a} \pm 5.1$ (58)	n.d.
	10 µM 1-MCP	$31.7^{a} \pm 4.2$ (25)	$33.8^{a} \pm 1.8$ (26)	$63.7^{b} \pm 3.6$ (49)	n.d.
	30 µM Cd	$24.0^{b} \pm 1.4$ (18)	$21.8^{b} \pm 1.3$ (16)	$41.5^{cd} \pm 2.2$ (31)	$48.4^{c} \pm 1.8$ (36)
	Cd + 1-MCP	$31.5^{a} \pm 3.6$ (29)	$20.7^{b} \pm 2.7$ (19)	$52.9^{bc} \pm 2.1$ (48)	< 10* (< 5)
	3 µM Hg	$25.1^{b} \pm 3.4$ (31)	< 10* (< 5)	$31.2^{d} \pm 1.4$ (38)	$20.7^{a} \pm 0.6$ (25)
	Hg + 1-MCP	$37.9^{a} \pm 1.9$ (35)	$18.6^{b} \pm 2.2$ (17)	$39.4^{d} \pm 1.8$ (36)	$13.8^{b} \pm 0.6$ (13)
**24 h**	Control	$37.3^{ab} \pm 1.9$ (21)	$41.2^{a} \pm 4.2$ (23)	$101.9^{a} \pm 2.8$ (56)	n.d.
	10 µM 1-MCP	$43.8^{a} \pm 2.3$ (26)	$40.0^{a} \pm 4.1$ (24)	$85.3^{b} \pm 1.6$ (50)	n.d.
	30 µM Cd	$27.8^{c} \pm 1.1$ (12)	$30.3^{b} \pm 1.4$ (13)	$44.5^{e} \pm 2.1$ (18)	$138.7^{c} \pm 7.0$ (57)
	Cd + 1-MCP	$35.9^{b} \pm 2.4$ (16)	$36.8^{ab} \pm 1.7$ (16)	$64.1^{c} \pm 1.1$ (29)	$88.1^{b} \pm 0.9$ (39)
	3 µM Hg	$27.5^{c} \pm 2.1$ (15)	$24.2^{d} \pm 0.4$ (13)	$53.4^{f} \pm 0.4$ (29)	$77.4^{b} \pm 3.1$ (42)
	Hg + 1-MCP	$35.1^{b} \pm 1.6$ (18)	$33.7^{b} \pm 1.1$ (17)	$72.2^{d} \pm 1.3$ (37)	$53.6^{a} \pm 5.5$ (28)

*A peak was detected, but concentration was below 10 nmol g^{-1} FW.
n.d.: not detected.

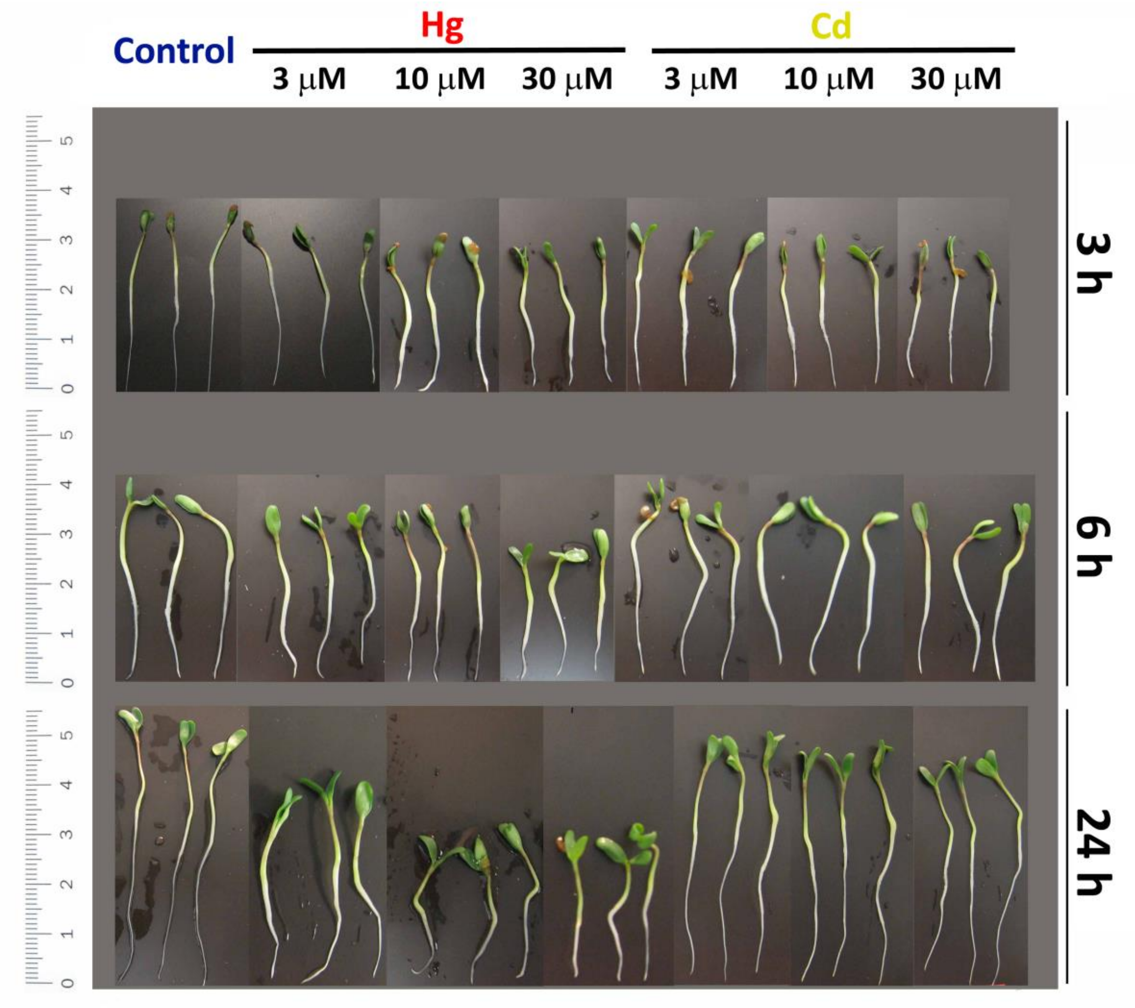

Supplementary Figure S1. Growth of alfalfa seedlings grown in the *microscale* system with Cd or Hg (0, 3, 10, 30 μM) for 3, 6 and 24 h. The millimetric scale on the left allows comparison of sizes in each treatment.

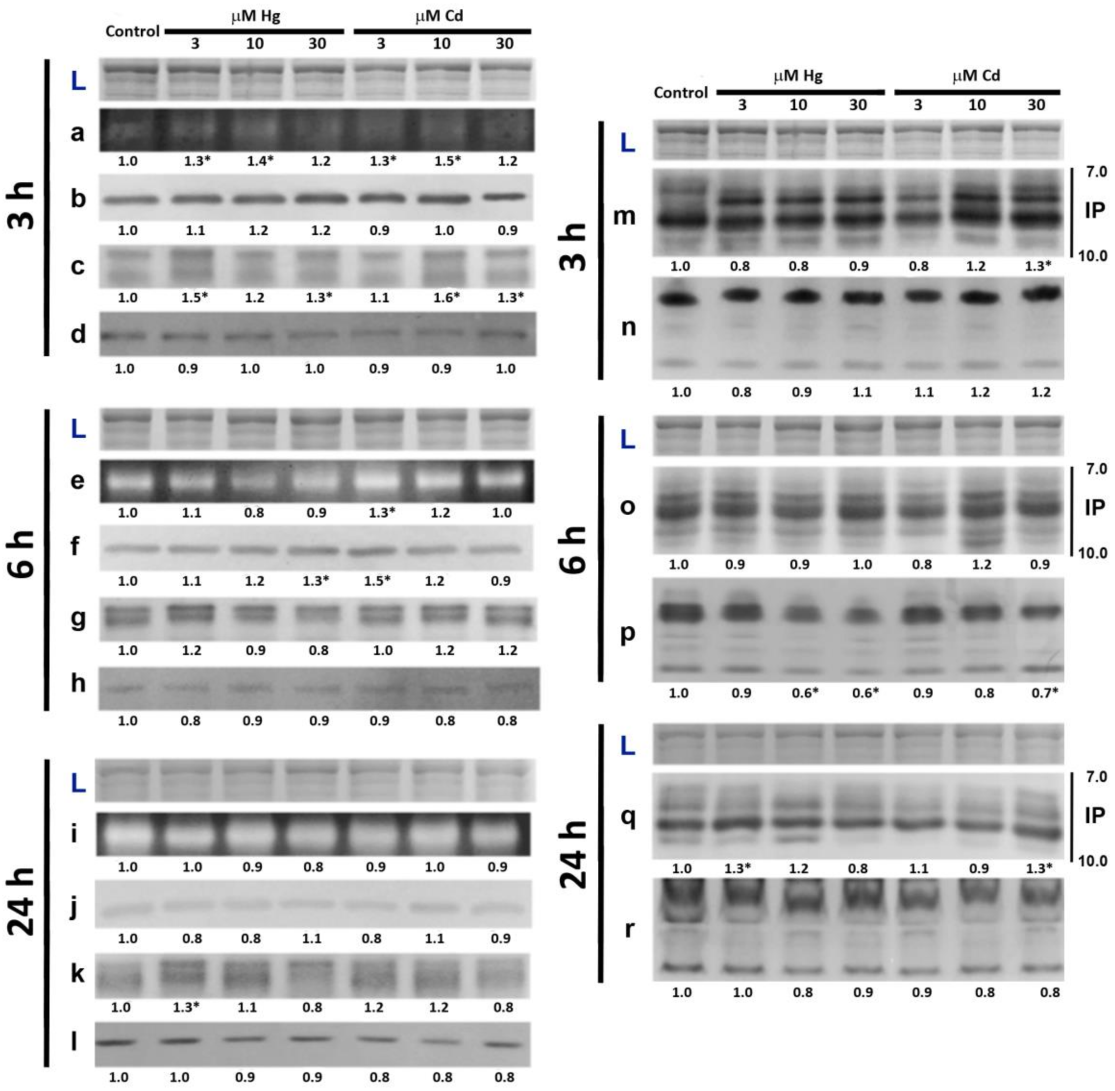

Supplementary Figure S2. Cotyledons redox enzymatic activities determined *in gel* after ND-PAGE, of alfalfa seedlings treated with 0, 3, 10 and 30 μM Hg or Cd for 3, 6 and 24 h. Identification of each band labelled with lower case letters: APX activity (a, e and i), α-APX immunodetection (b, f and j)(28 KDa), GR activity (c, g and k), α-GR immunodetection (d, h and l)(60 KDa), alkaline POX after isoelectric focusing at the immobilised pH (IP) 7.0 to 10.0 range (m, o and q), and plasmalemma NADPH-oxidase (n, p and r). Band intensity was normalised against Coomassie blue general protein staining after denaturing PAGE, corresponding to L (protein loading) bands. The numbers represent the fold change relative to control samples, and the asterisks mark fold-changes greater than 30%.

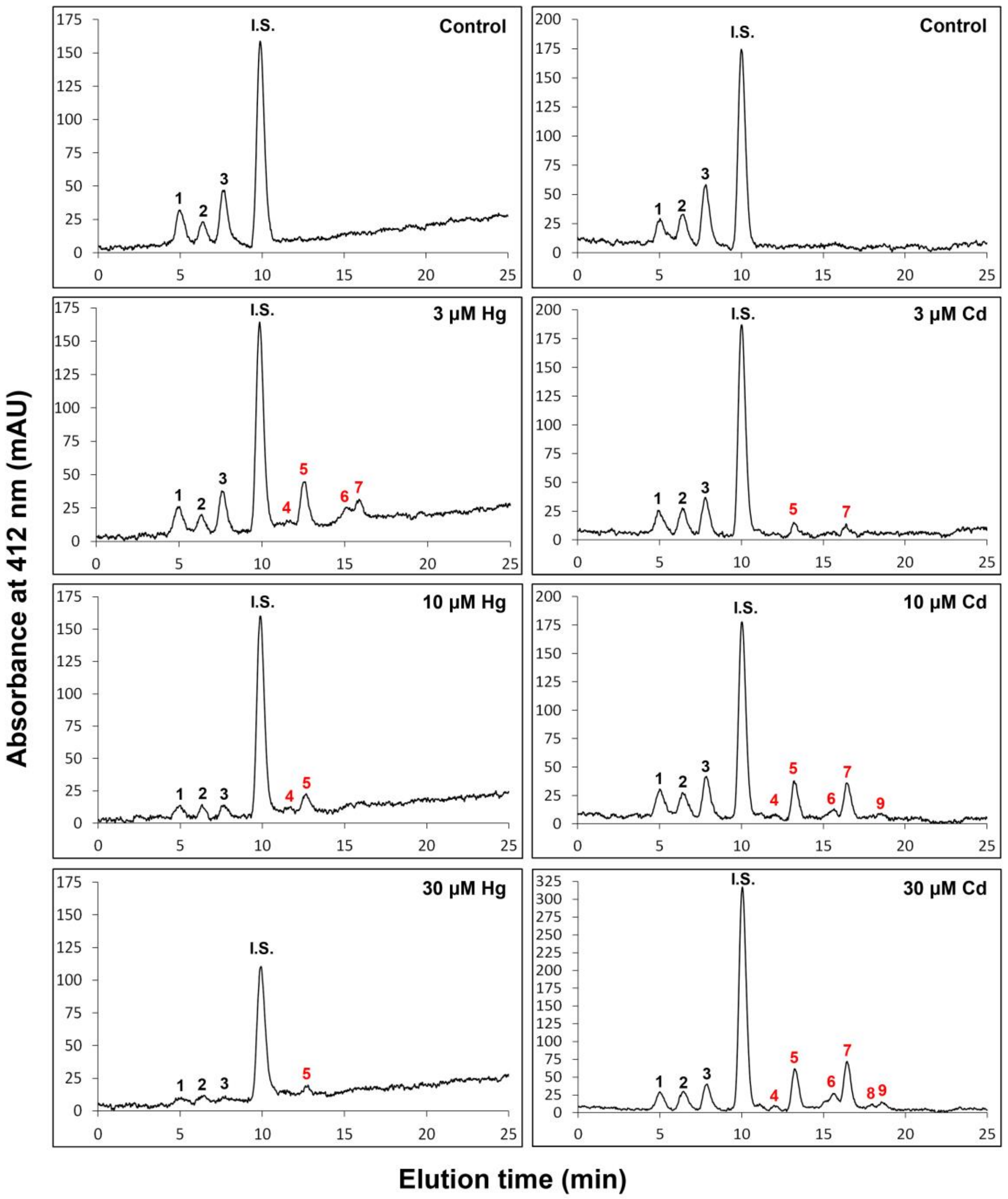

Supplementary Figure S3. Biothiols' chromatograms of extracts obtained from roots of alfalfa seedlings treated with Hg and Cd (0, 3, 10 and 30 μM) for 24 h. Black coloured peaks appeared in all samples: **1**, Cys; **2**, GSH; and **3**, hGSH. In red, peaks detected in seedlings exposed to Hg or Cd: **4**, PC_2 (-GluCys)$_2$-Gly); **5**, hPC_2 (-GluCys)$_2$-Ala); **6**, PC_3 (-GluCys)$_3$-Gly); **7**, hPC_3 (-GluCys)$_3$-Ala); **8**, PC_4 (-GluCys)$_4$-Gly); and **9**, hPC_4 (-GluCys)$_4$-Ala). I.S.: internal standard of N-acetyl cysteine (25 nmol per injection).
